# 2-Methyl-1,10b-dihydro-5*H*-pyrazolo[1,5-*c*][1,3]benzoxazin-5-one

**DOI:** 10.1107/S1600536809012173

**Published:** 2009-04-08

**Authors:** Viktor Kettmann, Jan Světlík

**Affiliations:** a Faculty of Pharmacy, Comenius University, Odbojarov 10, SK-83232 Bratislava, Slovakia

## Abstract

In the title compound, C_11_H_10_N_2_O_2_, a potential inhibitor of the cyclo­oxygenase-2 isoenzyme, the pyrazoline ring exists in a flat-envelope conformation while the puckering of the central oxazine ring is more severe. As a result, the mol­ecule as a whole is non-planar. The formal *sp* ^3^ pyrazoline N atom is *sp* ^2^ hybridized, with the lone-pair electrons delocalized through conjugation with the carbonyl group rather than the double bond of the pyrazoline ring.

## Related literature

For cyclo­oxygenase-2 (COX-2), see: Jahng *et al.* (2004 ▶); Ramatunge *et al.* (2004 ▶); Subbaramaiah *et al.* (2002 ▶). For bond parameters, see: Allen *et al.* (1987 ▶); Burke-Laing & Laing (1976 ▶). For background to the synthesis, see: Palomer *et al.* (2002 ▶); Světlík *et al.* (2005 ▶).
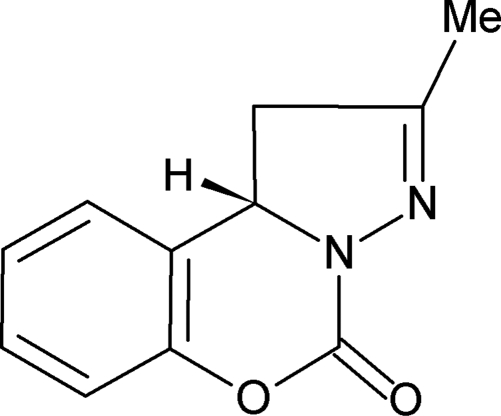


## Experimental

### 

#### Crystal data

C_11_H_10_N_2_O_2_
*M* *_r_* = 202.21
Orthorhombic, 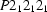

*a* = 7.240 (2) Å
*b* = 8.835 (2) Å
*c* = 15.755 (4) Å
*V* = 1007.8 (4) Å^3^
*Z* = 4
Mo *K*α radiation
μ = 0.09 mm^−1^
*T* = 296 K
0.30 × 0.25 × 0.20 mm

#### Data collection

Siemens P4 diffractometer
Absorption correction: none
2285 measured reflections
1674 independent reflections
1343 reflections with *I* > 2σ(*I*)
*R*_int_ = 0.021
3 standard reflections every 97 reflections intensity decay: none

#### Refinement

*R*[*F* ^2^ > 2σ(*F* ^2^)] = 0.055
*wR*(*F* ^2^) = 0.161
*S* = 0.96
1674 reflections
137 parameters
H-atom parameters constrained
Δρ_max_ = 0.26 e Å^−3^
Δρ_min_ = −0.22 e Å^−3^

### 

Data collection: *XSCANS* (Siemens, 1991 ▶); cell refinement: *XSCANS*; data reduction: *XSCANS*; program(s) used to solve structure: *SHELXS97* (Sheldrick, 2008 ▶); program(s) used to refine structure: *SHELXL97* (Sheldrick, 2008 ▶); molecular graphics: *PLATON* (Spek, 2009 ▶); software used to prepare material for publication: *SHELXL97*.

## Supplementary Material

Crystal structure: contains datablocks global, I. DOI: 10.1107/S1600536809012173/tk2391sup1.cif

Structure factors: contains datablocks I. DOI: 10.1107/S1600536809012173/tk2391Isup2.hkl

Additional supplementary materials:  crystallographic information; 3D view; checkCIF report

## Figures and Tables

**Fig. 1.**
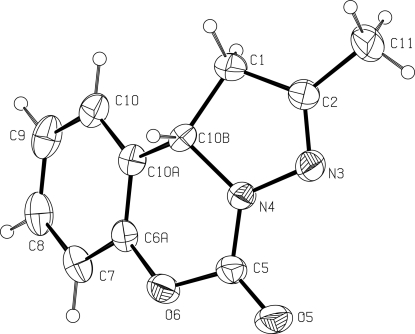
Displacement ellipsoid plot of (I) with the labelling scheme for the non-H atoms, which are drawn with displacement ellipsoids at the 35% probability level.

## supplementary crystallographic information

### Comment

Recently, as part of our on-going project aimed at developing new therapeutic agents, we focused our attention on 2-pyrazoline derivatives, which are known to possess cyclooxygenase-2 (COX-2) inhibitory activity (Jahng *et al.*, 2004), a feature which is of importance in treatment of inflammation (Ramatunge *et al.*, 2004) and cancer (Subbaramaiah *et al.*, 2002). In an effort to develop more potent and selective COX-2 inhibitors, we prepared a series of 2- and 5-substituted derivatives containing the tricyclic system featured in the title compound, (I), which still incorporates the putative COX-2 pharmacophore (Palomer *et al.*, 2002). Thus, the main aim of this work was to establish the spatial distribution of the pharmacophoric elements, *viz*. the hydrophobic groups and H-bond acceptors, which are responsible for binding of a compound to the COX-2 enzyme. To achieve this, we selected the title 2-methyl derivative, (I), for a single-crystal X-ray analysis.

The most interesting feature of (I), Fig. 1, is the spatial relationship between the pharmacophoric groups which is determined by the conformation of the (partially) saturated rings. Thus, the pyrazoline ring adopts a flat-envelope conformation with atom C10B as the flap; the deviation of the out-of-plane atom from the mean plane of the remaining four atoms is 0.334 (6) Å. The central six-membered ring is also non-planar and is puckered in such a manner that the four atoms O6, C6A, C10A and C10B are planar to within 0.004 (2) Å, while atoms N4 and C5 are displaced by 0.696 (5) and 0.590 (6) Å, respectively, to the same side of this plane. As a result of the relatively severe puckering of the central ring, the molecule as a whole is non-planar but consists of two approximately planar segments: O6,C6A,C7,C8,C9,C10,C10A,C10B [r.m.s. deviation 0.014 (3) Å] and C10B,C1,C2,C11,N3,N4,C5,O5,O6 [r.m.s. deviation 0.112 (3) Å], folded about the O6···C10B line [dihedral angle 31.3 (1)°].

The N3—N4 and C2—N3 bonds have pure single- and double-bond character, respectively (Burke-Laing & Laing, 1976). Even though the N4 atom is not involved in conjugation with the pyrazoline double bond, it is *sp*^2^ hybridized with its lone-pair electrons delocalized through conjugation with the adjacent carbonyl function as shown by the N4—C5 bond length (1.332 (4) Å), which is comparable to that typically found for amides (Allen *et al.*, 1987).

### Experimental

The synthesis of the title compound, (I), has been described (Světlík *et al.*, 2005). In short, a solution of 4,5-dihydro-(2-hydroxyphenyl)-3-methyl-1*H*-pyrazole (0.35 g, 2 mmol) and *N*,*N*'-carbonyldiimidazole (0.36 g, 2.2 mmol) in benzene (15 ml) were refluxed for 200 mins. After removal of the solvent, the oily residue was dissolved in dichloromethane (25 ml), washed with 10% HCl, water and dried (MgSO_4_). The solution was then concentrated under reduced pressure to give (I) (90% yield; m.p. 433–434 K) as colourless crystals.

### Refinement

The H atoms were visible in difference maps and were subsequently treated as riding atoms with distances C—H = 0.93 Å (CH_arom_), 0.97 (CH_2_), 0.98 Å (CH) and 0.96 Å (CH_3_), and with *U*_iso_(H) set to 1.2 (1.5 for the methyl H atoms) times *U*_eq_(parent atom). In the absence of significant anomalous scattering effects, 370 Friedel pairs were averaged in the final refinement.

### Crystal data

**Table:**

C_11_H_10_N_2_O_2_	*D*_x_ = 1.333 Mg m^−^^3^
*M**_r_* = 202.21	Melting point: 433 K
Orthorhombic, *P*2_1_2_1_2_1_	Mo *K*α radiation, λ = 0.71073 Å
Hall symbol: P 2ac 2ab	Cell parameters from 20 reflections
*a* = 7.240 (2) Å	θ = 7–18°
*b* = 8.835 (2) Å	µ = 0.09 mm^−^^1^
*c* = 15.755 (4) Å	*T* = 296 K
*V* = 1007.8 (4) Å^3^	Prism, colourless
*Z* = 4	0.30 × 0.25 × 0.20 mm
*F*(000) = 424	

### Data collection

**Table:**

Siemens P4 diffractometer	*R*_int_ = 0.021
Radiation source: fine-focus sealed tube	θ_max_ = 30.0°, θ_min_ = 2.6°
graphite	*h* = −1→10
ω/2θ scans	*k* = −1→12
2285 measured reflections	*l* = −1→22
1674 independent reflections	3 standard reflections every 97 reflections
1343 reflections with *I* > 2σ(*I*)	intensity decay: none

### Refinement

**Table:**

Refinement on *F*^2^	Primary atom site location: structure-invariant direct methods
Least-squares matrix: full	Secondary atom site location: difference Fourier map
*R*[*F*^2^ > 2σ(*F*^2^)] = 0.055	Hydrogen site location: inferred from neighbouring sites
*wR*(*F*^2^) = 0.161	H-atom parameters constrained
*S* = 0.96	*w* = 1/[σ^2^(*F*_o_^2^) + (0.058*P*)^2^ + 0.7099*P*] where *P* = (*F*_o_^2^ + 2*F*_c_^2^)/3
1674 reflections	(Δ/σ)_max_ = 0.003
137 parameters	Δρ_max_ = 0.26 e Å^−^^3^
0 restraints	Δρ_min_ = −0.22 e Å^−^^3^

### Special details

**Table:**

Geometry. All e.s.d.'s (except the e.s.d. in the dihedral angle between two l.s. planes) are estimated using the full covariance matrix. The cell e.s.d.'s are taken into account individually in the estimation of e.s.d.'s in distances, angles and torsion angles; correlations between e.s.d.'s in cell parameters are only used when they are defined by crystal symmetry. An approximate (isotropic) treatment of cell e.s.d.'s is used for estimating e.s.d.'s involving l.s. planes.Least-squares planes (*x*,*y*,*z* in crystal coordinates) and deviations from them (* indicates atom used to define plane)- 6.4736 (0.0083) *x* + 3.9501 (0.0194) *y* + 0.3894 (0.0333) *z* = 1.1812 (0.0071)* 0.0059 (0.0012) C1 * -0.0108 (0.0022) C2 * 0.0108 (0.0022) N3 * -0.0060 (0.0012) N4 - 0.3338 (0.0055) C10BRms deviation of fitted atoms = 0.00877.0450 (0.0031) *x* - 0.1068 (0.0223) *y* + 3.6269 (0.0223) *z* = 1.0294 (0.0128)Angle to previous plane (with approximate e.s.d.) = 29.57 (0.16)* -0.0027 (0.0010) O6 * 0.0053 (0.0019) C6A * -0.0050 (0.0018) C10A * 0.0024 (0.0009) C10B -0.6963 (0.0054) N4 - 0.5900 (0.0063) C5Rms deviation of fitted atoms = 0.00417.0642 (0.0025) *x* - 0.1723 (0.0086) *y* + 3.4371 (0.0150) *z* = 0.9846 (0.0059)Angle to previous plane (with approximate e.s.d.) = 0.82 (0.08)* -0.0143 (0.0023) O6 * -0.0087 (0.0029) C6A * 0.0109 (0.0030) C7 * 0.0160 (0.0032) C8 * -0.0063 (0.0031) C9 * -0.0177 (0.0029) C10 * -0.0034 (0.0027) C10A * 0.0234 (0.0023) C10BRms deviation of fitted atoms = 0.0140- 6.2356 (0.0052) *x* + 4.4868 (0.0103) *y* - 0.2799 (0.0136) *z* = 1.4163 (0.0043)Angle to previous plane (with approximate e.s.d.) = 31.34 (0.08)* -0.2463 (0.0027) C10B * 0.1374 (0.0029) C1 * 0.0194 (0.0039) C2 * -0.0476 (0.0032) N3 * -0.0351 (0.0028) N4 * 0.0138 (0.0033) C5 * 0.1614 (0.0027) O6 * 0.0426 (0.0034) C11 * -0.0456 (0.0025) O5Rms deviation of fitted atoms = 0.1123
Refinement. Refinement of *F*^2^ against ALL reflections. The weighted *R*-factor *wR* and goodness of fit *S* are based on *F*^2^, conventional *R*-factors *R* are based on *F*, with *F* set to zero for negative *F*^2^. The threshold expression of *F*^2^ > σ(*F*^2^) is used only for calculating *R*-factors(gt) *etc*. and is not relevant to the choice of reflections for refinement. *R*-factors based on *F*^2^ are statistically about twice as large as those based on *F*, and *R*- factors based on ALL data will be even larger.

### Fractional atomic coordinates and isotropic or equivalent isotropic displacement parameters (Å^2^)

**Table:**

	*x*	*y*	*z*	*U*_iso_*/*U*_eq_	
C1	0.1064 (5)	0.4867 (4)	−0.11974 (19)	0.0540 (8)	
H1A	0.0197	0.5671	−0.1328	0.065*	
H1B	0.2107	0.4926	−0.1582	0.065*	
C2	0.0157 (5)	0.3341 (4)	−0.1229 (2)	0.0600 (9)	
N3	−0.0199 (4)	0.2741 (3)	−0.05137 (18)	0.0581 (7)	
N4	0.0479 (4)	0.3750 (3)	0.00991 (16)	0.0484 (6)	
C5	0.0201 (5)	0.3524 (4)	0.0925 (2)	0.0523 (8)	
O5	−0.0494 (5)	0.2447 (3)	0.12627 (15)	0.0761 (9)	
O6	0.0794 (4)	0.4709 (3)	0.14279 (14)	0.0583 (7)	
C6A	0.1009 (4)	0.6157 (3)	0.1075 (2)	0.0475 (7)	
C7	0.0800 (5)	0.7370 (4)	0.1622 (2)	0.0594 (9)	
H7	0.0532	0.7219	0.2193	0.071*	
C8	0.1000 (6)	0.8811 (4)	0.1297 (3)	0.0675 (11)	
H8	0.0873	0.9643	0.1654	0.081*	
C9	0.1389 (5)	0.9035 (4)	0.0445 (3)	0.0669 (11)	
H9	0.1507	1.0011	0.0230	0.080*	
C10	0.1601 (5)	0.7788 (4)	−0.0086 (3)	0.0569 (8)	
H10	0.1858	0.7931	−0.0659	0.068*	
C10A	0.1430 (4)	0.6339 (3)	0.0233 (2)	0.0431 (6)	
C10B	0.1680 (5)	0.4926 (3)	−0.02737 (18)	0.0429 (6)	
H10B	0.2971	0.4598	−0.0235	0.051*	
C11	−0.0392 (8)	0.2580 (7)	−0.2042 (3)	0.0990 (19)	
H11A	−0.0912	0.1605	−0.1918	0.149*	
H11B	0.0677	0.2458	−0.2396	0.149*	
H11C	−0.1290	0.3190	−0.2331	0.149*	

### Atomic displacement parameters (Å^2^)

**Table:**

	*U*^11^	*U*^22^	*U*^33^	*U*^12^	*U*^13^	*U*^23^
C1	0.060 (2)	0.0602 (18)	0.0414 (14)	−0.0014 (17)	0.0052 (15)	−0.0004 (14)
C2	0.062 (2)	0.071 (2)	0.0464 (16)	−0.011 (2)	0.0099 (16)	−0.0100 (16)
N3	0.0687 (18)	0.0535 (14)	0.0521 (14)	−0.0145 (16)	0.0099 (14)	−0.0113 (13)
N4	0.0602 (15)	0.0425 (12)	0.0424 (12)	−0.0082 (13)	0.0002 (12)	0.0010 (10)
C5	0.069 (2)	0.0450 (15)	0.0434 (14)	−0.0054 (17)	−0.0015 (16)	0.0022 (13)
O5	0.116 (2)	0.0590 (14)	0.0528 (13)	−0.0199 (17)	0.0073 (15)	0.0105 (12)
O6	0.0811 (18)	0.0495 (12)	0.0442 (11)	−0.0036 (13)	−0.0074 (13)	0.0021 (9)
C6A	0.0454 (15)	0.0438 (15)	0.0532 (17)	−0.0029 (14)	−0.0089 (14)	−0.0026 (13)
C7	0.058 (2)	0.0586 (19)	0.0614 (19)	0.0009 (18)	−0.0116 (17)	−0.0163 (17)
C8	0.061 (2)	0.0489 (18)	0.092 (3)	0.0013 (18)	−0.013 (2)	−0.023 (2)
C9	0.055 (2)	0.0353 (14)	0.110 (3)	−0.0010 (15)	−0.005 (2)	−0.0001 (18)
C10	0.0453 (16)	0.0542 (19)	0.071 (2)	−0.0042 (15)	−0.0013 (17)	0.0076 (18)
C10A	0.0356 (13)	0.0404 (13)	0.0533 (16)	0.0044 (12)	−0.0012 (13)	−0.0006 (12)
C10B	0.0420 (14)	0.0383 (13)	0.0484 (15)	−0.0037 (12)	0.0046 (13)	0.0062 (12)
C11	0.106 (4)	0.137 (4)	0.054 (2)	−0.050 (4)	0.016 (2)	−0.038 (3)

### Geometric parameters (Å, °)

**Table:**

C1—C2	1.501 (5)	C7—C8	1.380 (5)
C1—C10B	1.523 (4)	C7—H7	0.9300
C1—H1A	0.9700	C8—C9	1.385 (6)
C1—H1B	0.9700	C8—H8	0.9300
C2—N3	1.272 (4)	C9—C10	1.393 (5)
C2—C11	1.499 (5)	C9—H9	0.9300
N3—N4	1.403 (4)	C10—C10A	1.381 (4)
N4—C5	1.332 (4)	C10—H10	0.9300
N4—C10B	1.477 (4)	C10A—C10B	1.493 (4)
C5—O5	1.200 (4)	C10B—H10B	0.9800
C5—O6	1.381 (4)	C11—H11A	0.9600
O6—C6A	1.404 (4)	C11—H11B	0.9600
C6A—C10A	1.371 (4)	C11—H11C	0.9600
C6A—C7	1.383 (4)		
			
C2—C1—C10B	101.0 (3)	C7—C8—H8	119.6
C2—C1—H1A	111.6	C9—C8—H8	119.6
C10B—C1—H1A	111.6	C8—C9—C10	119.5 (3)
C2—C1—H1B	111.6	C8—C9—H9	120.3
C10B—C1—H1B	111.6	C10—C9—H9	120.3
H1A—C1—H1B	109.4	C10A—C10—C9	120.3 (3)
N3—C2—C1	115.7 (3)	C10A—C10—H10	119.8
N3—C2—C11	121.1 (4)	C9—C10—H10	119.8
C1—C2—C11	123.2 (3)	C6A—C10A—C10	118.8 (3)
C2—N3—N4	105.9 (3)	C6A—C10A—C10B	116.5 (3)
C5—N4—N3	121.6 (3)	C10—C10A—C10B	124.7 (3)
C5—N4—C10B	125.7 (3)	N4—C10B—C10A	107.7 (2)
N3—N4—C10B	112.3 (2)	N4—C10B—C1	100.6 (3)
O5—C5—N4	127.9 (3)	C10A—C10B—C1	120.3 (3)
O5—C5—O6	118.5 (3)	N4—C10B—H10B	109.2
N4—C5—O6	113.6 (3)	C10A—C10B—H10B	109.2
C5—O6—C6A	119.9 (2)	C1—C10B—H10B	109.2
C10A—C6A—C7	122.4 (3)	C2—C11—H11A	109.5
C10A—C6A—O6	121.0 (3)	C2—C11—H11B	109.5
C7—C6A—O6	116.6 (3)	H11A—C11—H11B	109.5
C8—C7—C6A	118.2 (4)	C2—C11—H11C	109.5
C8—C7—H7	120.9	H11A—C11—H11C	109.5
C6A—C7—H7	120.9	H11B—C11—H11C	109.5
C7—C8—C9	120.8 (3)		
			
C10B—C1—C2—N3	14.6 (4)	C8—C9—C10—C10A	−0.2 (6)
C10B—C1—C2—C11	−168.2 (4)	C7—C6A—C10A—C10	−1.9 (5)
C1—C2—N3—N4	−2.2 (4)	O6—C6A—C10A—C10	179.1 (3)
C11—C2—N3—N4	−179.4 (4)	C7—C6A—C10A—C10B	177.9 (3)
C2—N3—N4—C5	174.6 (4)	O6—C6A—C10A—C10B	−1.2 (4)
C2—N3—N4—C10B	−12.4 (4)	C9—C10—C10A—C6A	1.5 (5)
N3—N4—C5—O5	5.9 (7)	C9—C10—C10A—C10B	−178.3 (3)
C10B—N4—C5—O5	−166.1 (4)	C5—N4—C10B—C10A	−40.0 (4)
N3—N4—C5—O6	−173.5 (3)	N3—N4—C10B—C10A	147.3 (3)
C10B—N4—C5—O6	14.4 (5)	C5—N4—C10B—C1	−166.7 (3)
O5—C5—O6—C6A	−158.0 (4)	N3—N4—C10B—C1	20.6 (3)
N4—C5—O6—C6A	21.5 (5)	C6A—C10A—C10B—N4	30.5 (4)
C5—O6—C6A—C10A	−28.5 (5)	C10—C10A—C10B—N4	−149.8 (3)
C5—O6—C6A—C7	152.4 (3)	C6A—C10A—C10B—C1	144.7 (3)
C10A—C6A—C7—C8	1.0 (5)	C10—C10A—C10B—C1	−35.6 (5)
O6—C6A—C7—C8	−180.0 (3)	C2—C1—C10B—N4	−19.1 (3)
C6A—C7—C8—C9	0.4 (6)	C2—C1—C10B—C10A	−137.0 (3)
C7—C8—C9—C10	−0.8 (6)		
